# Classical complement activation in light and heavy chain deposition disease with acquired cutis laxa and bronchiolitis obliterans: a case report of monoclonal gammopathy of clinical significance

**DOI:** 10.3389/fimmu.2025.1718342

**Published:** 2025-12-01

**Authors:** Yuxiong Chen, Yue Fan, Shoudong Yang, Haoyuan Cui, Wei Ye, Wenling Ye, Yubing Wen, Junling Zhuang, Ping Wang, Donglai Ma, Kai Fang, Peng Xia, Limeng Chen, Hang Li, Chao Li

**Affiliations:** 1 Department of Internal Medicine, Peking Union Medical College Hospital, Chinese Academy of Medical Sciences, Beijing, China; 2 Department of Cardiology, Peking Union Medical College Hospital, Peking Union Medical College and Chinese Academy of Medical Sciences, Beijing, China; 3 Department of Nephrology, Peking Union Medical College Hospital, Peking Union Medical College and Chinese Academy of Medical Sciences, Beijing, China; 4 Department of Hematology, Peking Union Medical College Hospital, Peking Union Medical College and Chinese Academy of Medical Sciences, Beijing, China; 5 Department of Respiratory and Critical Care Medicine, Peking Union Medical College Hospital, Peking Union Medical College and Chinese Academy of Medical Sciences, Beijing, China; 6 Department of Dermatology, Peking Union Medical College Hospital, Peking Union Medical College and Chinese Academy of Medical Sciences, Beijing, China

**Keywords:** case report, light and heavy chain deposition disease, acquired cutis laxa, bronchiolitis obliterans, classical complement activation

## Abstract

Light and heavy chain deposition disease (LHCDD) is a clonal plasma cell or monoclonal B-cell dyscrasia characterized by deposition of monoclonal immunoglobulin light and heavy chains. LHCDD mainly belongs to monoclonal gammopathy of renal significance (MGRS), including a spectrum of kidney disorders caused by a monoclonal protein (M-protein) secreted by a small plasma cell clone or other B-cell clones in patients who do not meet the diagnostic criteria for multiple myeloma or other B-cell malignancies. It may also occur as a renal complication of overt multiple myeloma. We report a 27-year-old man who presented clinically with chronic nephritic syndrome and was diagnosed with LHCDD confirmed by renal biopsy, accompanied by hypocomplementemia and bronchiolitis obliterans (BO). Notably, he initially developed acquired cutis laxa (CL) four years before renal dysfunction. Progressive dermatologic manifestations prompted repeat skin biopsies, revealing deposition of γ1 heavy chains, restrictive lambda light chains and complement components (C3, C4 and C1q) along dermal elastic fibers, establishing monoclonal gammopathy of dermatologic significance (MGODS) before systemic involvement. This case illustrates a rare constellation of MGRS, MGODS, and BO in a young adult and provides unique histologic and serologic evidence of classical complement pathway activation. Our findings support a potential immune-mediated mechanism underlying tissue injury in both renal and extrarenal manifestations of monoclonal gammopathy, highlighting the diagnostic value of early tissue biopsy and the importance of complement assessment in such cases.

## 1 Introduction

Light and heavy chain deposition disease (LHCDD) is a rare hematological disorder characterized by the deposition of nonamyloid monoclonal immunoglobulin light chains and heavy chains, presenting with proteinuria, hematuria, hypertension, and reduced glomerular filtration rate. The characteristics of LHCDD are the linear deposition of monoclonal light and heavy chains along the tubular and glomerular basement membranes, with Congo red staining being negative. Electron microscopy reveals punctate, amorphous, ground pepper-like deposits (1). The International Kidney and Monoclonal Gammopathy Research Group classified LHCDD within monoclonal gammopathy of renal significance (MGRS), including a spectrum of kidney disorders caused by a monoclonal protein (M-protein) secreted by a small plasma cell clone or other B-cell clones in patients who do not meet the diagnostic criteria for multiple myeloma or other B-cell malignancies (2). Previous studies have reported the potential ability of the heavy chain in activating complement (3, 4). Acquired cutis laxa (CL), an uncommon connective tissue disorder presenting as loose and redundant skin due to elastolysis, can also manifest in association with monoclonal gammopathies (5). Herein, we presented a rare case of LHCDD with generalized acquired CL and bronchiolitis obliterans. Renal and skin biopsies revealed hypocomplementemia and co-deposition of IgG-γ1 heavy chains, lambda light chains with complement components C3, C4 and C1q, suggesting that monoclonal immunoglobulin heavy chains may activate the classical complement pathway, contributing to complement-mediated renal injury and elastic fiber destruction.

## 2 Case presentation

A 27-year-old man presented with blurred vision, severe hypertension, and progressive renal impairment that began in January 2025 and was admitted to our hospital in February 2025 for further evaluation. He had a four-year history of acquired CL characterized by excessive wrinkling of the skin initially observed around the axillae, progressing to involve the trunk, neck, and face. Physical examination showed generalized skin laxity and mild periorbital edema. Laboratory tests at admission (**Table 1**) revealed mild anemia, hypoalbuminemia, renal impairment (serum creatinine 1.78 mg/dL), heavy proteinuria (3.39 g/day), microscopic hematuria, and hypocomplementemia (C354 mg/dL, reference 73-146; C49 mg/dL, reference 10-40). Further complement assays showed elevated serum C5b-9 (480 ng/mL; reference 75-219) and low CH50 (10 U/mL; reference 26-55), with normal factor B and H, indicating classical complement pathway activation. Serum IgA, IgG, and IgM were normal. Serum protein electrophoresis showed an M-spike (5.5%, 286 mg/dL). Serum immunofixation electrophoresis (IFE) identified a monoclonal IgG lambda band, with a markedly elevated serum lambda free light chain (47.5 mg/dL; reference 0.57-2.63), normal kappa light chain (1.72 mg/dL; reference 0.33-1.96), and a decreased kappa/lambda ratio (0.036; reference 0.26-1.65). Urine IFE confirmed monoclonal lambda light chain excretion. The hepatitis C virus (HCV) antibody was positive, but the HCV RNA was undetectable. Hepatitis B surface antigen, antinuclear antibody spectrum, antineutrophilic cytoplasmic antibody, rheumatoid factors, and cryoglobulin were negative. Bone marrow biopsy showed 0.5% plasma cells without light chain restriction. Multisystem evaluation showed no evidence of cardiac, hepatic, or neurologic involvement.

**Table 1:** Laboratory and pulmonary function findings at baseline and after five cycles of CyBorD therapy.

Variables	At admission	After the 1^st^ cycle	After the 2^nd^ cycle	After the 3^rd^ cycle	After the 4^th^ cycle	After the 5^th^ cycle
Laboratory tests						
Hb (g/dL)	9.3	10.6	12.3	11.8	13.3	13.2
Albumin (g/dL)	3.3	3.1	3.5	3.5	3.8	3.9
Cr (mg/dL)	1.78	1.71	1.80	1.30	0.98	1.10
eGFR (mL/min/1.73m^2^)	53.1	55.6	52.3	77.2	107.8	94.6
C3 (mg/dL)	54	54	62	67	62	73
C4 (mg/dL)	9	8	14	16	14	16
M-protein (%)	5.5	3.4	1.8	1.3	0	0
M-protein (mg/dL)	286	163	101	72	0	0
sFLC-λ (mg/dL)	47.5	9.02	7.17	3.06	2.14	1.6
sFLC-κ (mg/dL)	1.72	1.43	4.41	1.08	1.02	1.77
sFLC-κ/sFLC-λ	0.036	0.159	0.615	0.353	0.477	0.904
24h U-protein (g)	3.39	1.59	0.4	0.28	0.46	0.28
24h M-protein (mg)	15	14	0	0	0	0
Pulmonary function test						
FEV1 (%)	41	44.2	38	42	–	40
FVC (%)	79	77.6	77	77	–	83
FEV1/FVC (%)	43.7	47.8	41.2	54	–	49
PEF (%)	52	57.8	55	55	–	57
FEF25 (%)	22	22.3	17	21	–	18
FEF50 (%)	12	16.8	11	13	–	12
FEF75 (%)	7	14.2	9	8	–	8
MMEF75/25 (%)	10	15.3	–	12	–	10
RV/TLC (%)	44.6	50.0	46.3	50.3	–	49.1
DLCOc SB (%)	34	41.8	36	31	–	33
DLCOc/VA (%)	43	43.3	43	37	–	39

eGFR was calculated using the 2021 CKD-EPI formula. Abbreviations: CyBorD, cyclophosphamide, bortezomib and dexamethasone; C3, complement 3; C4, complement 4; CKD-EPI, Chronic Kidney Disease Epidemiology Collaboration; Cr, creatinine; DLCOc SB, corrected diffusion capacity of carbon monoxide measured by the single-breath method; DLCOc/VA, corrected diffusion capacity of carbon monoxide per liter of alveolar volume; eGFR, estimated glomerular filtration rate; FEF25, forced expiratory flow at 25% of forced vital capacity; FEF50, forced expiratory flow at 50% of forced vital capacity; FEF75, forced expiratory flow at 75% of forced vital capacity; FEV1, forced expiratory volume in one second; FVC, forced vital capacity; Hb, hemoglobin; MMEF75/25, maximal mid-expiratory flow between 25% and 75% of forced vital capacity; PEF, peak expiratory flow; RV, residual volume; sFLC, serum free light chain; TLC, total lung capacity.

**Figure 1:**
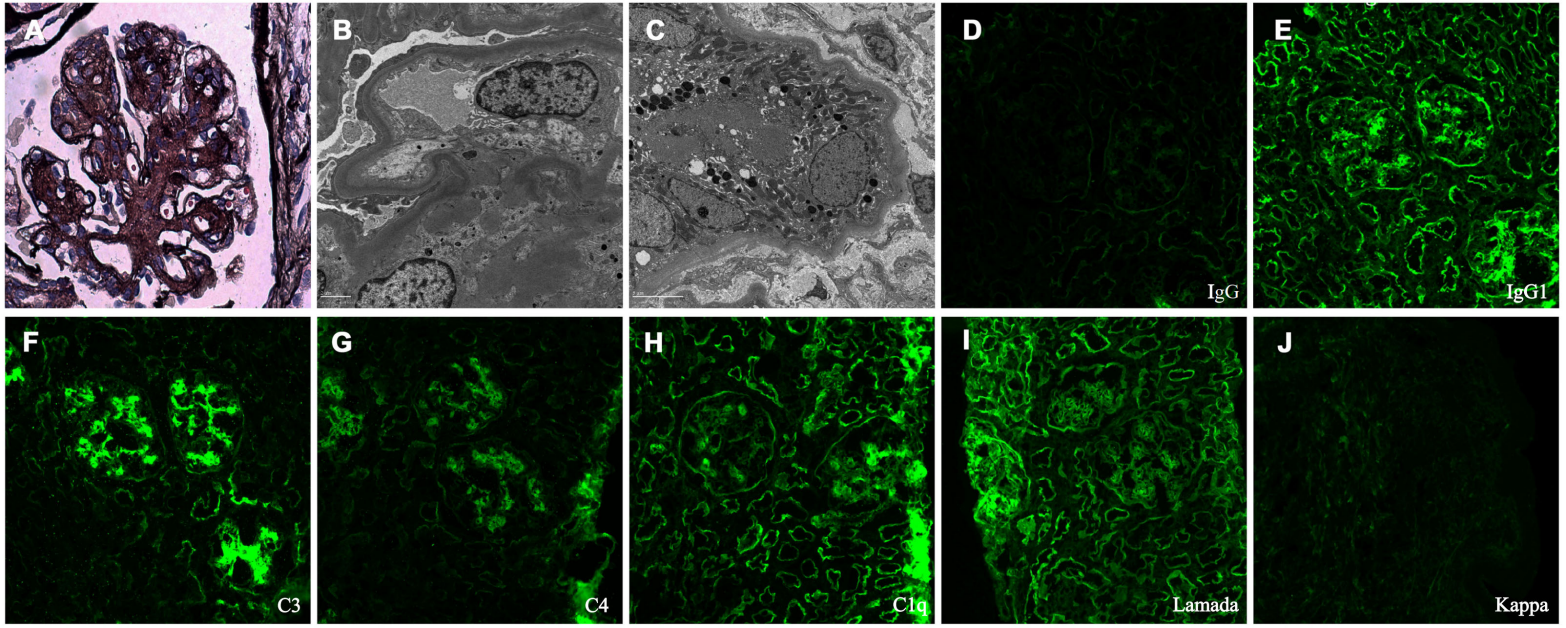
Renal biopsy findings. **(A)** Light microscopy of the kidney (periodic acid Schiff-methenamine silver, ×400) demonstrates nodular mesangial sclerosis with segmental double-contour formation of the glomerular capillary walls. **(B, C)** Electron microscopy of the kidney (×10,000) reveals powdery electron-dense deposits on the inner aspect of the glomerular basement membrane and the outer aspect of the tubular basement membrane. **(D-J)** Immunofluorescence staining of the kidney (direct immunofluorescence assay, ×200) shows strong granular mesangial deposition of C3, with weaker staining for C4 and C1q. Lambda light chain and IgG1 are positive along the mesangium and glomerular basement membranes. In the tubulointerstitial compartment, linear staining for C1q, IgG1 and lambda light chain is observed along the tubular basement membranes.

A kidney biopsy was performed three days after admission in February 2025. Light microscopy of the kidney biopsy revealed nodular mesangial sclerosis, segmental double-contour formation, and tubulointerstitial injury. Congo red staining was negative. Immunofluorescence demonstrated strong IgG1, C3 and lambda light chain deposition in glomeruli and tubular basement membranes, with weaker C4 and C1q staining. Electron microscopy revealed abundant finely granular electron-dense deposits (EDDs) within the mesangial matrix and along the inner aspect of the glomerular basement membrane, the outer surface of the TBM and peritubular capillary basement membranes (**Figure 1**). The kidney biopsy confirmed LHCDD and associated complement activation.

**Figure 2:**
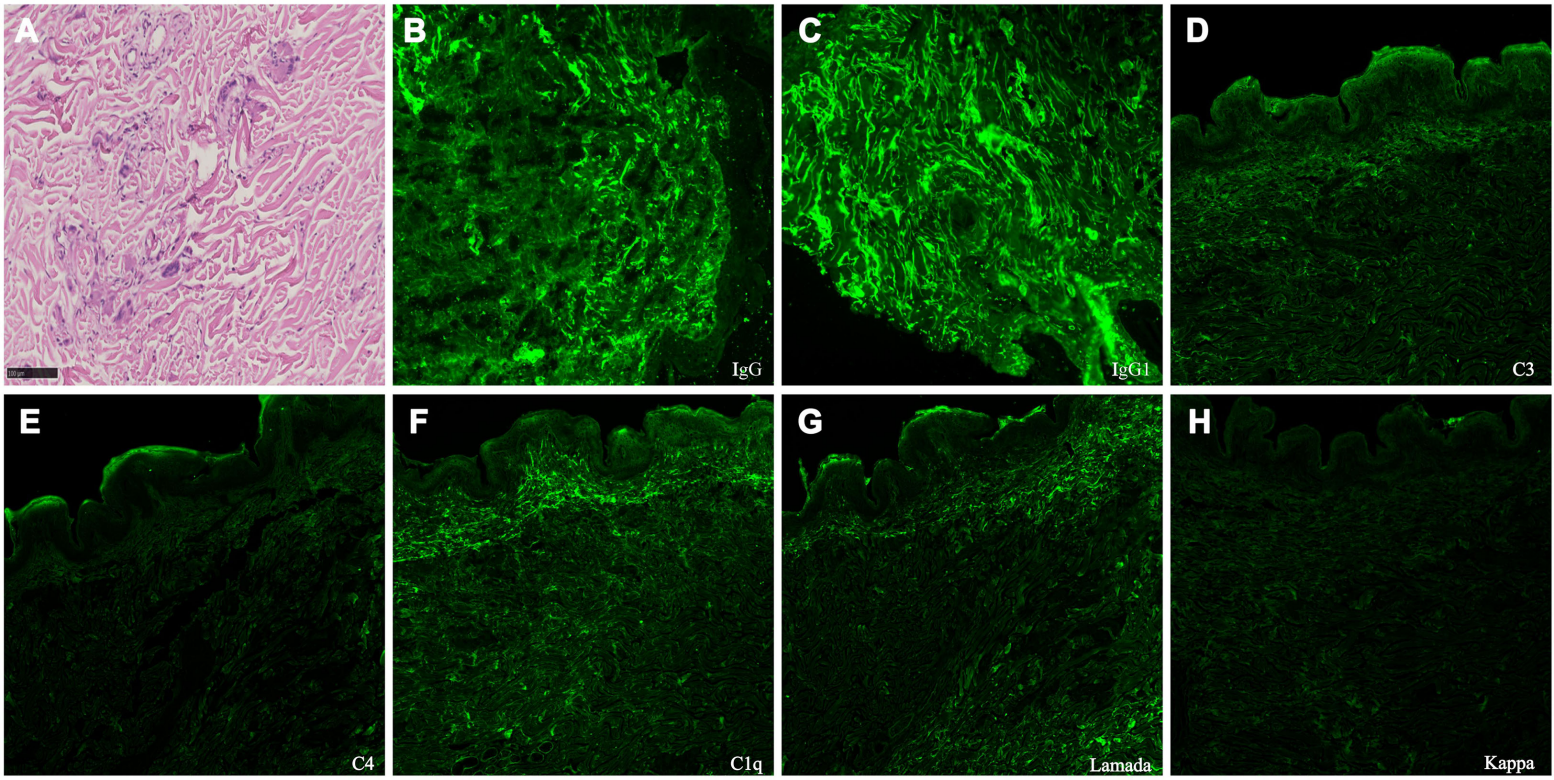
Skin biopsy findings. **(A)** Light microscopy of the skin (periodic acid-Schiff stain, ×400) demonstrates fragmentation and degeneration of dermal elastic and collagen fibers, predominantly in the mid to deep dermis. The surrounding dermis contains a granulomatous inflammatory infiltrate composed of lymphocytes, histiocytes, epithelioid macrophages, and multinucleated giant cells, consistent with elastolytic giant cell granulomas. **(B-H)** Direct immunofluorescence of the abdominal skin biopsy (×200) demonstrates IgG1, lambda light chain, C3, C4, and C1q deposited along dermal elastic fibers, consistent with the characteristics of monoclonal gammopathy of dermatologic significance (MGODS).

A skin biopsy in 2021 initially revealed elastolytic granulomas without immunostaining. Despite multiple immunomodulatory therapies and a facial rhytidectomy in 2024, laxity worsened. A repeat biopsy of the abdominal skin in February 2025 revealed elastolytic giant cell granulomas with co-deposition of IgG1, lambda light chain, C3, C4, and C1q, indicating monoclonal gammopathy of dermatologic significance (**Figure 2**). As for his progressive shortness of breath on exertion, pulmonary function testing showed obstructive ventilatory defect and reduced diffusion capacity (**Table 1**). Chest high-resolution CT demonstrated mosaic attenuation and expiratory bronchial collapse, consistent with bronchiolitis obliterans (BO).

The patient began his first cycle of cyclophosphamide, bortezomib and dexamethasone (CyBorD regimen) in March 2025 and completed five cycles by September 2025, resulting in a complete hematologic response and renal remission (**Table 1**). Serum complement C4 was normalized and C3 nearly recovered. However, CL and BO showed only mild symptomatic improvement (**Table 1**).

## 3 Discussion

LHCDD, a very rare form of monoclonal immunoglobulin deposition disease (MIDD), is classified alongside light chain deposition disease (LCDD) and heavy chain deposition disease (HCDD) (1). Acquired cutis laxa (CL) is a rare connective tissue disorder with heterogeneous etiologies, including inflammatory, drug-induced, and hematologic neoplasm–associated forms. When related to monoclonal gammopathy, it is referred to as monoclonal gammopathy of dermatologic significance (MGODS) (6). Most cases of acquired CL associated with monoclonal gammopathy were reported in multiple myeloma and occasionally in HCDD and LCDD (7). To our knowledge, this is the second reported case of LHCDD-associated acquired CL and the first with documented complement activation and bronchiolitis obliterans (BO) (8).

Complement activation is variably observed across MIDD subtypes (**Table 2**). The proportion of patients with low complement levels and renal complement deposition appears higher in HCDD compared with LCDD, suggesting that the heavy chain may play a more prominent role in driving complement activation (4, 10, 11). Furthermore, Bridoux et al. noted in their cohort that γ-heavy chain HCDD, particularly γ1 and γ3 subtypes, showed more frequent complement abnormalities, supporting the hypothesis that different heavy chain subtypes possess variable capacities to trigger classical complement pathway activation (4). The classical pathway is typically triggered when C1q binds to the Fc region of IgG or IgM within immune complexes, initiating downstream activation of C4 and C2. However, structural studies have shown that certain IgG subclasses, particularly IgG1 and IgG3, can self-assemble into hexameric arrays on target surfaces, allowing direct C1q binding and complement activation even in the absence of immune complexes (12, 13). This property may explain why our patient’s monoclonal IgG-γ1 heavy chain deposition efficiently engaged the classical pathway, as supported by co-deposition of C1q, C3, and C4 in renal and cutaneous tissues and hypocomplementemia with elevated sC5b-9.

**Table 2:** Reported complement abnormalities across MIDD subtypes in the literature.

Study	Disease	n	Low C3 (%)	Low C4 (%)	Glomerular C3 deposition (%)	Glomerular C4 deposition (%)
Wang et al., 2024, AJKD (9)	LHCDD	13	15	0	46	15
Zhang et al., 2020, AJKD (10)	HCDD	25	68	24	76	68
Bridoux et al., 2017, KI (4)	HCDD	15	33	13	53	33
Li et al., 2016, Ann Hematol (11)	LCDD	48	33	2.1	31	10

MIDD, monoclonal immunoglobulin deposition disease; C3, complement 3; C4, complement 4; AJKD, American Journal of Kidney Diseases; Ann Hematol, Annals of Hematology; KI, Kidney International; HCDD, heavy chain deposition disease; LHCDD, light and heavy chain deposition disease; LCDD, light chain deposition disease; n, number of patients.

The pathophysiology of acquired CL associated with monoclonal gammopathy remains unknown. Several cases of acquired CL associated with HCDD reported hypocomplementemia with co-deposits of heavy chain, C3 and C1q on kidney basement membranes and dermal elastic fibers (14, 15). In our patient, both renal and skin biopsies revealed co-deposition of IgG1, lambda light chains, and complement components (C1q, C3, and C4), alongside hypocomplementemia and elevated serum C5b-9, suggesting activation of the classical complement pathway (16). Importantly, serum C4 normalization and C3 recovery closely paralleled hematologic and renal responses to bortezomib-based therapy. These observations suggest that complement activation may function as a key amplifier, rather than a passive bystander process, of organ injury initiated by monoclonal immunoglobulin deposition via the classical pathway in our case. We acknowledge that complement activation was confirmed only after systemic involvement, as earlier complement data and skin immunostaining were unavailable. This limitation should be considered when interpreting its temporal relationship with monoclonal protein deposition.

In addition to renal and dermatologic involvement, our patient developed bronchiolitis obliterans, a form of small airway disease characterized by fixed airflow obstruction. The previous case of LHCDD with acquired CL only reported a one-year history of breathlessness at admission without the results of pulmonary function or chest CT scan (8). A retrospective national multicenter study in France reported 14 cases of acquired CL associated with monoclonal gammopathy. 8 cases presented pulmonary emphysema with chronic respiratory insufficiency requiring oxygen therapy in 4 cases and pulmonary transplantation in 2 cases (7). Another two cases of HCDD with acquired CL also presented with emphysema (14, 17). In patients with CL and monoclonal gammopathy, especially those with respiratory symptoms, high-resolution chest CT should be considered to assess for small airway disease. Given that elastic fibers are key structural components of both skin and lung tissue (18), systemic elastolysis triggered by complement-mediated inflammation may underlie this tri-organ involvement (17). The absence of a lung biopsy in this case limits direct histologic confirmation; however, the radiologic and functional findings, together with concurrent cutaneous and renal complement deposition, support the possibility of systemic complement-driven injury.

Owing to the rarity of LHCDD and the lack of randomized clinical trials, approved or standardized treatment options for LHCDD remain unclear. Bortezomib-based chemotherapy followed by autologous stem cell transplant seems to be an effective treatment option (19). Our patient received plasma cell-directed therapy with a complete hematologic and renal response but minimal improvement in skin or lung manifestations, consistent with previous reports that acquired CL and BO underlying monoclonal gammopathy rarely regress after hematologic control (7). The mechanisms of renal injury in MIDD mainly involve monoclonal immunoglobulin deposition causing direct basement membrane damage, complement activation, and secondary mesangial expansion and sclerosis, which may be reversible with early therapy. In contrast, acquired CL and BO reflect chronic elastolysis with irreversible elastic fiber degeneration. In our patient, the low renal chronicity score (3/12) indicated potential reversibility, whereas the four-year duration of CL suggested long-standing, irreversible elastic fiber loss. BO likely represents a similar process of persistent elastin damage.

In summary, we describe a rare case of LHCDD accompanied by acquired CL and BO, with clinicopathologic evidence of classical complement activation. The findings suggest that CL may precede systemic involvement and indicate complement-mediated tissue injury. Early recognition of monoclonal gammopathy is essential for improving outcomes, underscoring the need for comprehensive diagnostic evaluation in patients presenting with CL. This case provides further insight into the potential role of complement dysregulation in systemic injury associated with monoclonal gammopathy, warranting future mechanistic studies.

## Data Availability

The original contributions presented in the study are included in the article/**Supplementary Material**. Further inquiries can be directed to the corresponding authors.
